# *Manta birostris*, predator of the deep? Insight into the diet of the giant manta ray through stable isotope analysis

**DOI:** 10.1098/rsos.160717

**Published:** 2016-11-30

**Authors:** Katherine B. Burgess, Lydie I. E. Couturier, Andrea D. Marshall, Anthony J. Richardson, Scarla J. Weeks, Michael B. Bennett

**Affiliations:** 1 School of Biomedical Sciences, Planning and Environmental Management, The University of Queensland, St Lucia, Queensland 4072, Australia; 2 Centre for Applications in Natural Resource Mathematics, Planning and Environmental Management, The University of Queensland, St Lucia, Queensland 4072, Australia; 3 Biophysical Oceanography Group, School of Geography, Planning and Environmental Management, The University of Queensland, St Lucia, Queensland 4072, Australia; 4 Marine Megafauna Foundation, Truckee, CA, USA; 5 CSIRO Oceans and Atmosphere, EcoSciences Precinct, GPO Box 2583, Dutton Park, Queensland 4001, Australia; 6 Laboratoire des Sciences de l'Environnement Marin, IUEM, rue Dumont d'Urville, Université de Bretagne Occidentale, UMR 6539 LEMAR (IRD/UBO/CNRS/Ifremer), Plouzané 29280, France

**Keywords:** diet, eastern tropical Pacific Ocean, mesopelagic, trophic ecology, elasmobranch, planktivore

## Abstract

The characterization of diet for the giant manta ray *Manta birostris* has been problematic given their large-scale movement patterns and the difficulty in obtaining stomach contents from this species. The large majority of existing information is based on observational data limited to feeding events at the sea surface during daylight. Recently discovered aggregation sites for the giant manta ray off mainland Ecuador are some of the most accessible to date and provide a unique opportunity for researchers to gather much needed information on this elusive species. To assess how important surface zooplankton is to giant manta ray diet, we conducted stable isotope analysis (^15^N and ^13^C) on *M. birostris* muscle and surface zooplankton. Trophic position estimates placed *M. birostris* overall at a secondary consumer level of approximately 3.4 but there was large variation in *δ*^15^N and *δ*^13^C values among individuals. *Manta birostris* muscle tissue *δ*^13^C values were also not consistent with this species feeding predominantly on surface zooplankton and suggest that the majority of dietary intake is of mesopelagic origin. Given the conservative life history and fisheries pressure on large planktivores, knowledge of their trophic role and foraging strategies is essential to better understand their ecology and develop effective conservation measures.

## 1. Background

Manta rays are large filter feeding elasmobranchs, but despite considerable study many aspects of their biology and ecology remain enigmatic. Dietary information for *Manta* species is based mostly on observational data, primarily gained from near-surface feeding events during daylight. Zooplankton collected by plankton tows at the time of these events has been assumed to represent the species' diet.

Non-lethal, minimally invasive biochemical methods, such as bulk stable isotope analysis (SIA), have proved useful in the examination of dietary intake of large, mobile and difficult-to-observe elasmobranch species [1,2]. The ratio of heavy to light isotopes of carbon (*δ*^13^C) and nitrogen (*δ*^15^N) can provide information on dietary sources [3] and trophic position, respectively [4]. Carbon isotopes are transferred conservatively through the food web with initial source variations in the aquatic environment dependent on the extent of mixing of inorganic carbon. For example, considerable variability in *δ*^13^C exists between benthic and surface water marine algae, and consumers of benthic carbon sources are enriched in ^13^C compared with pelagic surface feeders [5]. As ^14^N is lost more rapidly than ^15^N during the processes of metabolism and excretion, increasing values of *δ*^15^N are found as animals attain higher trophic positions [6].

For diet reconstructions using SIA data, Bayesian mixing models can be used to determine prey source contributions to the isotopic composition of a consumer tissue [7]. Proportional contributions of *n* + 1 different sources, where *n* is the number of isotopes being measured in the study, can be measured using these mixing models [8]. Mixing models can provide a mean solution of dietary inputs, along with minimum and maximum estimates, where the latter are sometimes the more robust output from the model [9]. While use of mixing models comes with considerable limitations, they provide the only way to glean quantitative/semi-quantitative dietary composition data from SIA values. Although conclusions about distinct dietary contributions from prey categories cannot occur without *a priori* knowledge of dietary habits for a given species, SIA is a useful approach particularly for species where stomach contents analysis (SCA, which can provide high-resolution dietary information) may be inappropriate or may yield unrepresentative results due to differential prey residency times in the gut [10].

Here, using SIA, we present information on the feeding ecology of *Manta birostris* in the eastern equatorial Pacific along with novel insights into the origin of its main dietary sources.

## 2. Material and methods

Muscle tissue biopsies were collected from photographically identified manta rays with a 5 mm diameter biopsy punch mounted on a hand-spear, while on SCUBA. Sampling was conducted at Isla de la Plata (1.2786° S, 81.0686° W) and Bajo Copé (1.81706° S, 81.06362° W), Ecuador, during July–October, 2012–2014. Zooplankton was collected with a plankton net (200 µm mesh, 50 cm diameter) using horizontal near-surface tows. All muscle tissue biopsies and zooplankton samples were placed on ice immediately after collection and stored at −18°C until required for SIA.

Muscle samples were soaked in deionized water for 24–48 h to remove urea [11]. Manta ray muscle tissue and zooplankton samples were dried at 50–60°C for 24–48 h and then each was homogenized. A known mass (≈1.5 mg) of each sample was weighed, placed in a tin capsule and pelletized. Samples were analysed for *δ*^13^C and *δ*^15^N using an isotope ratio mass spectrometer (Hydra 20–22; Sercon Ltd, UK) coupled with an elemental analyser (Europa EA-GSL; Sercon Ltd, UK).

Stable isotope ratios were measured relative to two internationally recognized standards; Vienna Pee Dee Belemnite limestone for C^13^/C^12^ and atmospheric air for N^15^/N^14^ [12]. Two additional internal standards of ammonium sulfate and sucrose were used in each run. Results are expressed in delta (*δ*) notation in parts per thousand (‰) as follows:

2.1 $$\delta^{H}X(‰)=\left(\frac{R_{\mathrm{sample}}}{R_{\mathrm{standard}}}-1\right)\times 1000,$$

where *X* is the element, *H* denotes the heavy isotope mass number and *R* is the ratio of heavy-to-light isotopes. Temporal, inter- and intra-specific differences in bulk *δ*^13^C and *δ*^15^N values for *M. birostris* and surface zooplankton were assessed using two-way ANOVAs with a type I error rate of *α* = 0.05. Throughout, results are presented as mean and standard deviation unless otherwise stated.

Lipid removal was deemed unnecessary given the majority of *M. birostris* C : N ratios were less than 3.5 [13]. The zooplankton C : N ratio was 4.3 ± 0.5, thus *δ*^13^C values were normalized using an arithmetic correction for zooplankton lipids [14]:

2.2 $$\delta^{13}C_{\mathrm{LN}}=\delta^{13}C_{\mathrm{BULK}}+7.95\left(\frac{\left(\text{C}:{\text{N}}_{\mathrm{BULK}}-3.8\right)}{\text{C}:{\text{N}}_{\mathrm{BULK}}}\right),$$

where _LN_ is the *δ*^13^C value after lipid normalization and _BULK_is the non-normalized *δ*^13^C or C : N value.

Relative trophic positions using *M. birostris* isotopic data were calculated using [15]:

2.3 $$T_{\mathrm{LSIA}}=\left(\frac{\left(\delta^{15}{\text{N}}_{\mathrm{consumer}}-\delta^{15}{\text{N}}_{\mathrm{primary}}\right)}{\text{DTDF}}\right)+2.5,$$

where *T*_LSIA_ is the relative trophic level, *δ*^15^N_consumer_ is the average isotopic value for *M. birostris* tissue. To account for spatial and temporal heterogeneity in baseline values, the *δ*^15^N_primary_ used (7‰) was the average *δ*^15^N value of surface zooplankton that was collected at Isla de la Plata during 2013–2014 and mesopelagic fish species collected from the North Pacific Subtropical Gyre (NPSG) in 2009–2011 [16]. An integer value of 2.5 was used, as surface zooplankton tows and mesopelagic fish species comprised a mixture of primary (*T*_L_ = 2) and secondary consumers (*T*_L_ = 3). Estimates of *T*_LSIA_ are sensitive to assumptions about the trophic fractionation of *δ*^15^N. Therefore, two *T*_LSIA_ estimates were generated for *M. birostris* using elasmobranch specific *δ*^15^N diet tissue discrimination factors (DTDFs): 2.3‰ [17] and 3.7‰ [18].

A Bayesian mass-balance mixing model assessed the contribution of different sources to the diet of *M. birostris* in the R package ‘simmr’ [19,20]. Bayesian inference was used to address natural variation and uncertainty of stable isotope data to generate probability distributions of source contributions as percentages of total diet. Source, consumer and trophic enrichment factor variability was incorporated into the model. Co-occurring turtles, yellowfin tuna and thresher sharks were not included in the mixing model as the number of source contributions needed to also assess the diet of all of these species *M. birostris* would have surpassed the number of isotopes +1.

There are no demersal, benthic or deep-sea bulk stable isotope values available for zooplankton from coastal Ecuador and, unfortunately, due to logistical constraints we could not sample mesopelagic zooplankton from the region. Instead, sources for all mixing models were constrained to surface zooplankton from Isla de la Plata and assumed representative of mesopelagic sources from other studies. There is strong isotopic similarity between mesopelagic zooplankton and mesopelagic fishes [21], therefore, small mesopelagic fish (*Cyclothone alba* (*n* = 3), *Cyema atrum* (*n* = 3) and *Hygophum proximum* (*n* = 5)) from the NPSG with equivalent trophic positions to primary and secondary copepod consumers (2.1–2.9 [22]) were used as a representative offshore mesopelagic food source. Overall mean *δ*^13^C and *δ*^15^N values from small mesopelagic fish species (*Cyclothone alba*, *Cyema atrum* and *H. proximum*) were −17.6 ± 0.8‰ and 6.2 ± 1.5‰, respectively [16]. Surface zooplankton were from Isla de la Plata, coastal Ecuador (*n* = 35 net hauls) and had non-normalized lipid values for *δ*^13^C and *δ*^15^N of −20.5 ± 0.6‰ and 7.8 ± 1‰, respectively. The lipid-normalized value for surface zooplankton *δ*^13^C was −19.7 ± 1‰.

There are no experimentally determined diet tissue discrimination factors for manta rays or other large planktivorous elasmobranch species. Therefore, separate mixing models were run incorporating experimentally determined DTDFs from other elasmobranch species: *Triakis semifasciata* (1.7 ± 0.5 for *δ*^13^C and 3.7 ± 0.4 for *δ*^15^N [18]) and large pelagic sharks *Carcharias taurus* and *Negaprion brevirostris* (0.9 ± 0.33 for ^13^C and 2.29 ± 0.22 for ^15^N [17]). To account for the uncertainty in appropriate DTDF values and lipid-normalized of surface zooplankton *δ*^13^C four separate mixing models were run. Model 1 source inputs comprised mesopelagic fishes and lipid-normalized surface zooplankton *δ*^13^C values along with DTDFs from large sharks [17]. Model 2 source inputs were mesopelagic fishes and non-lipid-normalized surface zooplankton *δ*^13^C with the large shark DTDF [17]. Models 3 and 4 comprised the same source inputs as models 1 and 2, respectively, but used DTDF values from *T. semifasciata* [18]. To determine an overall estimate of the mean contribution to the diet of *M. birostris* from mesopelagic and surface sources, the mean source contribution for surface and mesopelagic prey from the four mixing models was averaged.

For inferences on species-interactions and the structure of communities using biochemical analyses, it is helpful to place the focus species into context with other co-occurring species [23]. The isotopic niche can be a powerful way to investigate the ecological niche of an animal because its chemical composition is influenced by what it consumes [24]. The isotopic niche structure of other vertebrates that seasonally co-occur with *M. birostris* in the eastern tropical Pacific Ocean and for which isotopic information is available was compared. This was done within a Bayesian framework using 95% credible intervals between species groups and stable isotope Bayesian ellipses in R package ‘SIBER’ [25]. These vertebrates included marine turtles (olive ridley *Lepidochelys olivacea*, green *Chelonia mydas* and loggerhead *Caretta caretta* [26]), yellowfin tuna *Thunnus albacares* [27], and the pelagic thresher shark *Alopias pelagicus* [28].

## 3. Results

For *M. birostris* muscle tissue mean *δ*^13^C and *δ*^15^N values were −16.8 ± 1.1‰ and 10.6 ± 1.5‰, respectively. Male (*n* = 45) and female (*n* = 30) *δ*^13^C values were indistinguishable from each other and across sampling years (two-way ANOVA: *F*_3,62_ = 1.924; *p* = 0.13) (table 1). *δ*^15^N values were not affected by sex (*p* = 0.35) but significantly differed between biopsies taken in 2014 and 2012 and between 2014 and 2013 (two-way ANOVA: *F*_3,62_ = 16.27; *p *< 0.05) (Tukey's HSD, *p* < 0.05). The average trophic position estimates for *Manta birostris* elasmobranch specific *δ*^15^N DTDFs of 3.7‰ and 2.3‰ was 3.4 (range 2.5–4.6) and 3.7 (range 2.3–5.6), respectively.

**Table 1.** Mean (±s.d.) *δ*^13^C and *δ*^15^N values for *Manta birostris*.

sample	*N*	C : N ± s.d.	*δ*^13^C ± s.d.	*δ*^15^N ± s.d.
*Manta birostris* (Ecuador)	75	3.3 ± 0.3	−16.8 ± 1.1	10.6 ± 1.5
male	45	3.3 ± 0.3	−17.0 ± 1.1	10.7 ± 1.3
female	30	3.3 ± 0.4	−16.6 ± 1.1	10.5 ± 1.7
2012	26	3.1 ± 0.2	−16.4 ± 1.2	11.2 ± 1.2
2013	27	3.1 ± 0.3	−17.1 ± 1.1	11.4 ± 1.1
2014	22	3.5 ± 0.3	−16.8 ± 0.9	9.3 ± 1.0

There was no difference in the isotopic composition of surface zooplankton when manta rays were feeding (*n* = 4) or not feeding (*n* = 31) (*δ*^13^C, *p* = 0.237) (*δ*^15^N, *p* = 0.975). As was the case for *M. birostris*, surface zooplankton isotopic composition differed among sampling years (*δ*^13^C, two-way ANOVA: *F*_2,32_ = 16.38; *p* < 0.05) (*δ*^15^N, two-way ANOVA: *F*_2,32_ = 14.45; *p* < 0.05).

**Figure 1.**
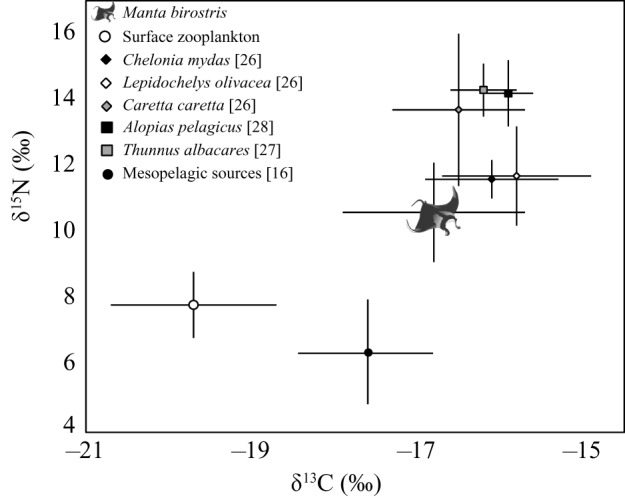
Mean *δ*^15^N and *δ*^13^C values for *M. birostris*, surface zooplankton (*δ*^13^C lipid normalized), mesopelagic sources and other co-occurring large vertebrates from Ecuador and the broader eastern equatorial Pacific region. Error bars represent standard deviation.

**Figure 2.**
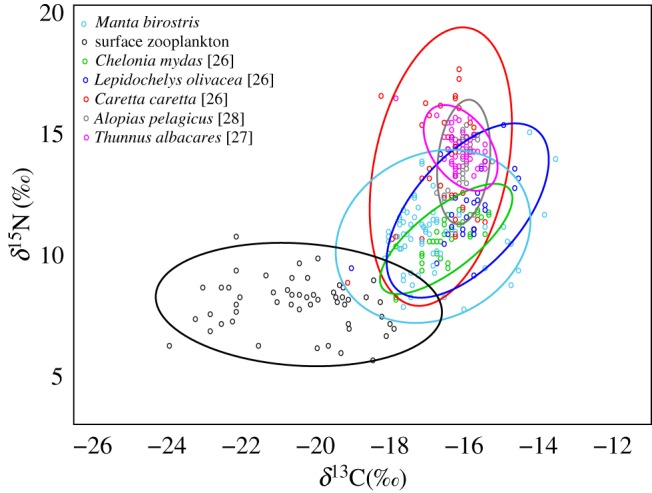
Bi-plot of *δ*^15^N and *δ*^13^C values with Bayesian ellipses overlaid for co-occurring organisms from the eastern equatorial Pacific Ocean.

Average enrichment between *M. birostris* and surface zooplankton (lipid normalized) sampled off mainland Ecuador was 2.9‰ and 2.8‰ for *δ*^13^C and *δ*^15^N values, respectively (figure 1). When surface zooplankton *δ*^13^C was not normalized the enrichment between zooplankton and *M. birostris δ*^13^C was 3.7‰. There was a high degree of overlap in isotopic niche space between *M. birostris* and other co-occurring vertebrates from the eastern equatorial Pacific (figure 2).

**Figure 3.**
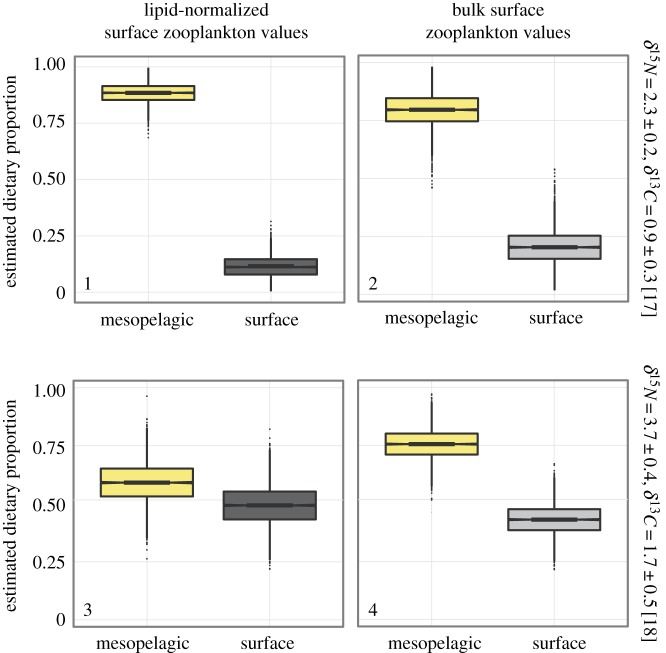
Box plots of Bayesian stable isotope mixing models for surface zooplankton (light grey), lipid-normalized surface zooplankton (LN) (dark grey) and mesopelagic (yellow) prey dietary source contributions to *Manta birostris*. Model number is pictured in the bottom left corner of each panel. On the secondary *Y-*axis is the DTDF from large shark species [17] used in models 1 and 2 and the DTDF from *T. semifasciata* [18] used for models 3 and 4. The central box spans the 2.5–97.5% confidence intervals with the middle line denoting the median.

From the four mixing models generated, surface zooplankton and mesopelagic sources were found on average to contribute 27% and 73% to the diet of *M. birostris*, respectively (table 2). All mixing models found mesopelagic sources to generate a majority contribution in comparison to surface zooplankton to the diet of *M. birostris* (figure 3; electronic supplementary material, table S3). The highest estimated source contribution for surface zooplankton to the diet of *M. birostris* was 43%, which was still lower than the most conservative estimate for mesopelagic source contribution (57%) (Model 3). Model 3, which used an elasmobranch specific *δ*^15^N DTDF of 3.7% and lipid-normalized surface zooplankton *δ*^13^C values, had the highest credible interval overlap of dietary contributions from mesopelagic sources (45–64%) and surface zooplankton (32–57%) (figure 3; electronic supplementary material, table S3).

**Table 2.** Mean (± s.d.) source contributions of surface zooplankton and mesopelagic sources to *M. birostris* diet from four mixing models. Model 1 source inputs comprised of mesopelagic fishes and lipid-normalized surface zooplankton *δ*^13^C values and used DTDFs from large sharks [17]. Model 2 source inputs were mesopelagic fishes and non-lipid-normalized surface zooplankton *δ*^13^C with the large shark DTDF [17]. Models 3 and 4 comprised the same source inputs as models 1 and 2, respectively, but used DTDF values from *T. semifasciata* [18]. Also shown is the mean source contribution calculated from all four mixing models.

source	model 1	model 2	model 3	model 4	mean
surface zooplankton	0.21 ± 0.07	0.12 ± 0.04	0.43 ± 0.06	0.33 ± 0.04	0.27 ± 0.14
mesopelagic sources	0.79 ± 0.07	0.88 ± 0.04	0.57 ± 0.06	0.68 ± 0.04	0.73 ± 0.14
s.d. *δ*^13^C	0.87 ± 0.17	0.79 ± 0.16	0.80 ± 0.13	0.84 ± 0.13	0.82 ± 0.04
s.d. *δ*^15^N	2.0 ± 0.25	2.05 ± 0.27	1.06 ± 0.19	0.95 ± 0.22	1.51 ± 0.58

## 4. Discussion

### 4.1. Manta ray feeding ecology

Large differences in *δ*^13^C and *δ*^15^N values indicate there is a broad range of dietary intake and habitats occupied by individual *M. birostris* off mainland Ecuador. Here, average *M. birostris δ*^13^C values were not consistent with this species feeding predominantly on surface zooplankton and suggest a larger reliance on mesopelagic food sources. Our SIA results placed *M. birostris* at a relative trophic position similar to that of other mobulids; *Mobula mobular* (3.6) [29], *Mobula thurstoni* (3.3) [30] and *Manta alfredi* (3) [31], confirming that they are all at least secondary consumers. There are no quantitative estimates of dietary composition for *M. birostris* and difficulties exist in assigning pre-2009 observations of manta ray feeding to the species involved due to the taxonomic revision of the genus *Manta* in 2009 [32]. Current knowledge of manta ray diet is based on accounts of feeding on a variety of abundant surface zooplankton by both recognized species, a small number of studies using biochemical analyses of tissue samples and a description of preserved stomach contents from an individual *M. alfredi* collected in 1935 [31,33–35].

There was large within-population isotopic niche variability of *M. birostris δ*^13^C values, which probably reflects available prey *δ*^13^C composition within occupied habitats. Planktivory as a feeding strategy has evolved independently in many vertebrate groups, including whales [36], sharks and teleosts [37]. In addition, adaptive radiation of planktivorous megafauna during the cenzoic era was concurrent with changes in global climate, which included increased productivity along with amplified patchiness of marine systems [38]. While *M. birostris* is considered a generalist carnivore, large differences in *δ*^13^C occur among individuals. This could be indicative of individual specialists within this subpopulation, which would facilitate a reduction in con-specific competition for resources within a patchy oceanic environment.

### 4.2. *δ*^13^C enriched food source origin

Average enrichment of *δ*^13^C between *M. birostris* and surface zooplankton was approximately three times higher than the literature values for mixed fish species (0.4 ± 1.3‰ (mean ± s.d.) [15]) and large sharks (0.9 ± 0.3‰ [17]). Similar results were found in a separate study for *M. alfredi*, which also had enriched *δ*^13^C values compared with its presumed surface zooplankton prey [31]. Surface zooplankton has been considered a primary food source for manta rays, based on numerous observations of foraging behaviour at the surface during daylight hours [39,40]. However, all mixing models estimated that mesopelagic sources comprised the majority of dietary intake for *M. birostris*. Electronic tag studies on *M. birostris*, *M. alfredi* and closely related *Mobula tarapacana* have shown that these rays, despite being predominantly surface dwellers, dive to depths of approximately 1400 m (A. Marshall 2010, unpublished data), 432 and 2000 m, respectively [41,42]. The dive-profiles suggest that all of these species forage at depth in the deep scattering layers. In addition, video of *M. birostris* taken at depth with a submersible vehicle confirms that individuals forage on mesopelagic sources in the Mexican east Pacific region [43]. The results from the mixing model in this study are consistent with the idea that this submersible footage may be indicative of a common event.

Large overlap in isotopic niche between *M. birostris* and all other marine vertebrates sampled from the eastern equatorial Pacific was found, and although isotopic niche is not the same as ecological niche, it can be used to infer characteristics of community structure and niche breadth of community members [25]. Apart from the loggerhead turtle, individuals of which transverse the entirety of the tropical Pacific in their lifetime [44], *M. birostris* has the broadest isotopic niche compared with other co-occurring marine vertebrates examined in the eastern equatorial Pacific Ocean. Additionally, expected enrichments in *δ*^13^C and *δ*^15^N for large shark species [17] occurred between *M. birostris*, a secondary consumer, and the thresher shark; a tertiary consumer that typically inhabits the upper regions of the water column but obtains the majority of its diet from the mesopelagic environment [28]. These expected enrichments indicate that both of these elasmobranch species are feeding within the same mesopelagic food web.

Mesopelagic prey occurs in much cooler temperatures than those in surface waters. It is thus expected that an ectotherm, such as manta ray, would exhibit compensatory behaviour for body heat loss after foraging on those prey in cold waters. Studies on large ectothermic planktivores such as the sunfish and the whale shark showed behavioural indications of deep feeding, with time spent within mesopelagic depth involving body temperature decrease always followed by a recovery time in warmer surface waters [45,46]. Manta rays are commonly seen in surface waters or cleaning in shallow coral reef habitats typically in tropical or subtropical regions [40,47]. Off mainland Ecuador, *M. birostris* aggregate around cleaning stations at Isla de la Plata, which is situated less than 40 km from a continental shelf edge that descends to approximately 3000 m [48]. It is possible that a driver behind aggregative of *M. birostris* at this site relates to individuals undergoing thermal recovery in warm surface waters after foraging at depth nearby.

### 4.3. Variability in isotopic baselines

Inter-annual variability in SI-values (^13^C and ^15^N) was found for both manta rays and surface zooplankton, with a large range between individual *M. birostris δ*^15^N values (7.3‰). Natural variation in consumer *δ*^15^N can be a consequence of a change in dominant primary producers at the base of a food web [49]. Such baseline shifts can influence food-chain length, which affects relative trophic position estimates for wide-ranging predators that forage in isotopically different oceanic regions [50]. Yellowfin tuna occurring at higher latitudes had higher *δ*^15^N values, which is consistent with *δ*^15^N values spatial variation of particulate organic matter within the eastern equatorial Pacific region [49]. Therefore, it is possible that high *δ*^15^N values of some *M. birostris* reflect large home ranges that comprise Ecuadorian, and higher latitude waters that are characterized by higher primary producer and subsequent zooplankton *δ*^15^N values [49]. However, these recorded baseline changes occurred in surface waters, and surface zooplankton are suspected not to comprise a majority of dietary intake for *M. birostris* in this region. It is currently unknown if vertical baseline changes at depth are similar to or coincide with horizontal baseline changes in surface waters in the eastern equatorial Pacific.

While primary producer baseline changes probably account for some of the variation in *δ*^15^N values and trophic position of *M. birostris*, the targeting of higher trophic level prey both in surface waters and at depth may also be a factor in high *δ*^15^N values seen in this study. *Manta birostris* has distinctive filtering pads and this species could capture and consume relatively large prey via a dead-end sieving mechanism [51,52]. While the diets of *Manta* spp. are poorly characterized, there are reports of individuals feeding on zooplankton and small to moderate sized fish [53]; however, it is uncertain to which species these observations relate. Diets of other large marine planktivores are better known and typically comprise both macroscopic zooplankton and higher trophic level prey. Whale sharks have been observed to feed on mysid and sergestid shrimps and ‘bait fish’ when in continuous ram-feeding mode, which is analogous to manta ray feeding mode [54,55]. Additionally, baleen whales (fin *Balaenoptera physalus*, common minke *Balaenoptera acutorostrata* and humpback *Megaptera novaeangliae*) eat a variety of macroscopic zooplankton along with many species of schooling fishes [56–58]. To differentiate between the various mechanisms contributing to isotopic variation in *M. birostris* in the eastern tropical Pacific, more data are needed on the isotopic composition of available prey across larger horizontal and vertical areas where individuals are likely to feed.

### 4.4. Limitations

A potential limitation of this study is the assumption that mesopelagic isotope values from the NPSG are representative of mesopelagic sources, offshore of mainland Ecuador and the broader eastern equatorial Pacific region, which *M. birostris* of this subpopulation feeds. There might be expected differences in isotopic values as the NPSG is less productive than the coastal upwelling Eastern Boundary Current (EBC) system off Ecuador, where a shallow thermocline facilitates enhanced nutrient supply [59]. Small average differences in *δ*^13^C (0.7‰) have been found between mesopelagic fishes collected in the NPSG and another EBC (California), with larger differences occurring in *δ*^15^N (8.5‰) [60]. However, different species were collected between these two sites and there were considerable differences in maximum sizes of mesopelagic fish sampled from California (490 mm) compared with those collected from the NSPG (277 mm). Additionally, mean bulk trophic position estimates for mesopelagic fish collected from the NSPG (2.2) were much lower than those for California (3.8) and this could have been attributed to the observed large difference in *δ*^15^N values between the two regions [60]. In another study, there was no predictable difference in *δ*^15^N values of northern fur seals that forage inshore (California) or offshore (140–180° W, NPSG); however, northern fur seals are not mesopelagic feeders [61]. There is no current consensus whether isotopic values would differ between mesopelagic sources in the NSPG and off mainland Ecuador given that there is no data for the latter.

### 4.5. Future work

While stomach contents analysis can provide good quantitative descriptions of diet [35], in the case of manta rays (listed as Vulnerable on the IUCN's Red List), such approaches should be restricted to situations where rays have suffered natural mortality or have been landed in commercial and artisanal fisheries. In such circumstances, SCA should be used to help validate and test the findings of SIA. However, the low resolution of bulk SIA precludes detailed dietary assessment from this technique alone, and critical values such as isotopic incorporation rate and diet tissue discrimination factors need to be determined experimentally for planktivorous elasmobranchs to aid interpretation of SIA results. In addition, better biochemical characterization of potential prey sources, such as mesopelagic and demersal zooplankton, over various spatio-temporal scales would assist in interpretations of unexpected biochemical profiles of consumers. However, collecting information on low- and mid-trophic prey communities is challenging and current direct sampling methods are not adequate to provide enough representative samples. Instead, we can use suitable values gleaned from the literature and focus on marine top predators to monitor the health of pelagic food webs as their feeding ecologies and spatial distributions provide a direct insight into food web dynamics, oceanic productivity and critical megafauna habitats [61].

### 4.6. Conclusion

Manta rays and other giant planktivorous elasmobranchs can provide high socio-economic benefits through ecotourism and may also have an important ecological role as a concentrated food drop to the deep as carcasses [62,63]. This study, along with others, suggests that manta rays and other large planktivorous elasmobranchs that live in these low latitude patchy marine systems, need to be energetically subsidized by mesopelagic resources [55]. The mesopelagic zone is the next frontier for open ocean fisheries [64], and it is concerning that we still do not fully understand the reliance on this zone by marine megafauna that already face threats in well characterized surface habitats [47].

## Supplementary Material

Supplementary information for sample collection, isotope values for individual Manta birostris and surface zooplankton tows, and mixing model summary statistics.Included here is a detailed sample collection protocol for obtaining M. birostris muscle tissue samples from free swimming animals. Additionally, the CN isotope values for individual Manta birostris and surface zooplankton tows are presented, along with mixing model summary statistics for the mean credible interval source contributions of surface zooplankton and mesopelagic sources to M. birostris diet.

## Data accessibility

Stable isotope data for each individual manta ray and zooplankton tow has been made available in the electronic supplementary material attached to this publication.
