# Supplementary material for: Manta birostris, predator of the deep? Insight into the diet of the giant manta ray through stable isotope analysis
Source: R Soc Open Sci. 2016 Nov 30;3(11):160717. doi: 10.1098/rsos.160717 (PMC5180158; doi:10.1098/rsos.160717)
Supplement: Supplementary information for sample collection, isotope values for individual Manta birostris and surface zooplankton tows, and mixing model summary statistics.Included here is a detailed sample collection protocol for obtaining M. birostris muscle tissue samples from free swimming animals. Additio [file rsos160717supp1.docx]

***Manta birostris*, predator of the deep? Insight into the diet of the giant manta ray through stable isotope analysis**

Katherine B. Burgess, Lydie I.E. Couturier, Andrea D. Marshall, Anthony J. Richardson, Scarla J. Weeks and Michael B. Bennett

**Supplementary Material**

1. **Supplementary Methods**

**Sample Collection**

- 1. Giant manta ray muscle samples were acquired from free-swimming and photographically identified individuals using a pole-spear with a biopsy punch fitted on the end. Biopsies were obtained from the same part of the manta (left wing, outside the body cavity) (Fig. S1).


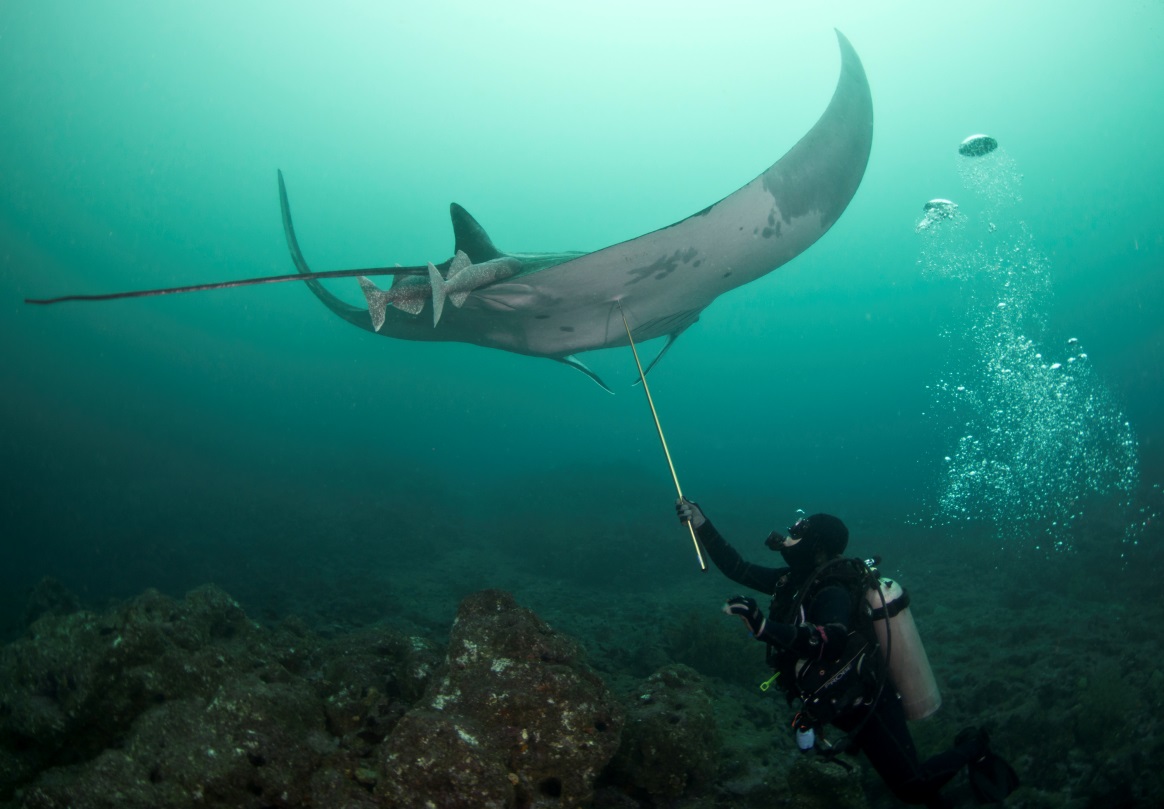


Figure S1. Biopsy being taken from a giant manta ray at Isla de la Plata, Ecuador.

- 1. Horizontal surface tows to collect zooplankton were conducted using a 200µm mesh plankton net (50cm diameter) for 5 minutes at an average speed of 1.5-2 knots.
  2. Both manta ray and zooplankton samples were placed immediately on ice after collection and once back on-shore were frozen at -18°C until required for stable isotope analysis.
  3. Co-occurring thresher shark and marine turtle samples from other studies that were used for qualitative isotopic comparisons with *M. birostris* in this current study underwent lipid extraction procedures. Thresher shark C:N ratios were 3.1 whereas C:N ratios were not reported for marine turtle species [28, 26]. Lipid extraction was not conducted on yellowfin tuna muscle, but this species is known to predominantly have low C:N ratios. In another study from the Indian Ocean reported C:N was 3.2 with 15/245 individuals samples having C:N values that surpassed 3.5 (Ménard et al., 2007).

1. **Supplementary Results**
   1. Manta ray and zooplankton δ^13^C, δ^13^C_normalised_ and δ^15^N values were used to construct the mixing model (table S1 and table S2).

Table S1. Individual giant manta ray C:N, δ^13^C, δ^15^N and trophic position estimates. TP_1_ refers to trophic position estimate generated from a δ^15^N DTDF of 3.7‰ [17], TP_2_ estimates were generated using a δ^15^N DTDF of 2.3‰ [16].

| Date Sampled | Species | Sex | C:N | δ^13^C | δ^13^C _normalised_^*^ | δ^15^N | TP_1_ | TP_2_ |
| --- | --- | --- | --- | --- | --- | --- | --- | --- |
| 13/09/2012 | *Manta birostris* | Female | 2.8 | -14.8 | -14.8 | 10.4 | 3.4 | 3.7 |
| 17/09/2012 | *Manta birostris* | Female | 3.3 | -18.1 | -18.1 | 9.7 | 3.2 | 3.4 |
| 17/09/2012 | *Manta birostris* | Female | 3.2 | -16.7 | -16.7 | 11.2 | 3.6 | 4.1 |
| 19/09/2012 | *Manta birostris* | Female | 3.3 | -17.1 | -17.1 | 10.4 | 3.4 | 3.7 |
| 21/09/2012 | *Manta birostris* | Female | 3.5 | -17.1 | -17.1 | 13.6 | 4.3 | 5.1 |
| 23/09/2012 | *Manta birostris* | Female | 3.4 | -16.5 | -16.5 | 12.1 | 3.9 | 4.5 |
| 25/09/2012 | *Manta birostris* | Female | 3.1 | -15.4 | -15.4 | 11 | 3.6 | 4.0 |
| 28/09/2012 | *Manta birostris* | Female | 3.1 | -17.1 | -17.1 | 13.7 | 4.3 | 5.2 |
| 29/09/2012 | *Manta birostris* | Female | 3.1 | -16.8 | -16.8 | 11.3 | 3.7 | 4.1 |
| 3/10/2012 | *Manta birostris* | Female | 2.7 | -14.7 | -14.7 | 8.6 | 2.9 | 2.9 |
| 5/10/2012 | *Manta birostris* | Female | 3.2 | -16.8 | -16.8 | 12.7 | 4.0 | 4.7 |
| 6/10/2012 | *Manta birostris* | Female | 3.3 | -17 | -17 | 10.8 | 3.5 | 3.9 |
| 7/10/2012 | *Manta birostris* | Female | 3.2 | -16.9 | -16.9 | 11.1 | 3.6 | 4.0 |
| 7/10/2012 | *Manta birostris* | Female | 2.7 | -13.5 | -13.5 | 13.8 | 4.3 | 5.2 |
| 11/09/2012 | *Manta birostris* | Male | 3.2 | -17.1 | -17.1 | 10.3 | 3.4 | 3.7 |
| 14/09/2012 | *Manta birostris* | Male | 3.3 | -17.5 | -17.5 | 11.6 | 3.7 | 4.3 |
| 19/09/2012 | *Manta birostris* | Male | 3.3 | -17 | -17 | 10.9 | 3.6 | 3.9 |
| 25/09/2012 | *Manta birostris* | Male | 2.7 | -15.4 | -15.4 | 9.3 | 3.1 | 3.3 |
| 27/09/2012 | *Manta birostris* | Male | 3.3 | -17.4 | -17.4 | 10.5 | 3.4 | 3.8 |
| 3/10/2012 | *Manta birostris* | Male | 2.7 | -13.8 | -13.8 | 11.5 | 3.7 | 4.2 |
| 3/10/2012 | *Manta birostris* | Male | 3.4 | -17.5 | -17.5 | 10.7 | 3.5 | 3.9 |
| 4/10/2012 | *Manta birostris* | Male | 3.4 | -17.3 | -17.3 | 11.4 | 3.7 | 4.2 |
| 5/10/2012 | *Manta birostris* | Male | 3 | -16.1 | -16.1 | 11.5 | 3.7 | 4.2 |
| 6/10/2012 | *Manta birostris* | Male | 3.3 | -17.1 | -17.1 | 11.1 | 3.6 | 4.0 |
| 6/10/2012 | *Manta birostris* | Male | 3.2 | -16.8 | -16.8 | 11.6 | 3.7 | 4.3 |
| 8/10/2012 | *Manta birostris* | Male | 3.3 | -17.4 | -17.4 | 11.1 | 3.6 | 4.0 |
| 3/09/2013 | *Manta birostris* | Female | 3.3 | -16.2 | -16.2 | 10.8 | 3.5 | 3.9 |
| 22/09/2013 | *Manta birostris* | Female | 3.3 | -16.7 | -16.7 | 11.3 | 3.7 | 4.1 |
| 2/09/2013 | *Manta birostris* | Male | 3.5 | -17.3 | -17.3 | 12.3 | 3.9 | 4.6 |
| 3/09/2013 | *Manta birostris* | Male | 3.5 | -17.7 | -17.7 | 11.1 | 3.6 | 4.0 |
| 3/09/2013 | *Manta birostris* | Male | 3.5 | -17.6 | -17.6 | 11.7 | 3.8 | 4.3 |
| 12/09/2013 | *Manta birostris* | Male | 3.4 | -18 | -18 | 10.4 | 3.4 | 3.7 |
| 18/09/2013 | *Manta birostris* | Male | 3.4 | -17.7 | -17.7 | 11.4 | 3.7 | 4.2 |
| 20/09/2013 | *Manta birostris* | Male | 3.4 | -18 | -18 | 10.8 | 3.5 | 3.9 |
| 22/09/2013 | *Manta birostris* | Male | 3.4 | -17.3 | -17.3 | 12.2 | 3.9 | 4.5 |
| 1/10/2013 | *Manta birostris* | Male | 3.4 | -17.2 | -17.2 | 11.8 | 3.8 | 4.3 |
| 2/10/2013 | *Manta birostris* | Male | 3.4 | -17.7 | -17.7 | 11.3 | 3.7 | 4.1 |
| 2/10/2013 | *Manta birostris* | Male | 3.4 | -17.6 | -17.6 | 10.7 | 3.5 | 3.9 |
| 4/10/2013 | *Manta birostris* | Male | 3.4 | -18 | -18 | 10.6 | 3.5 | 3.8 |
| 5/10/2013 | *Manta birostris* | Male | 3.6 | -17.3 | -17.1 | 11 | 3.6 | 4.0 |
| 6/10/2013 | *Manta birostris* | Male | 3.3 | -17.6 | -17.6 | 11.2 | 3.6 | 4.1 |
| 6/10/2013 | *Manta birostris* | Male | 2.9 | -17.7 | -17.7 | 10.1 | 3.3 | 3.6 |
| 6/10/2013 | *Manta birostris* | Male | 2.8 | -14.9 | -14.9 | 11 | 3.6 | 4.0 |
| 1/10/2013 | *Manta birostris* | Male | 2.6 | -14.2 | -14.2 | 14.9 | 4.6 | 5.7 |
| 22/09/2013 | *Manta birostris* | Male | 4.7 | -17.7 | -16.4 | 9.8 | 3.3 | 3.5 |
| 22/09/2013 | *Manta birostris* | Female | 3.8 | -18.3 | -17.9 | 8.5 | 2.9 | 2.9 |
| 1/10/2013 | *Manta birostris* | Male | 4.3 | -18.1 | -17.2 | 7.6 | 2.7 | 2.5 |
| 1/10/2013 | *Manta birostris* | Male | 5.1 | -18.4 | -16.7 | 9.3 | 3.1 | 3.3 |
| 2/10/2013 | *Manta birostris* | Male | 4.4 | -19.8 | -18.8 | 7.2 | 2.6 | 2.3 |
| 2/10/2013 | *Manta birostris* | Male | 4.6 | -18.9 | -17.7 | 7.4 | 2.6 | 2.4 |
| 2/10/2013 | *Manta birostris* | Male | 4.5 | -19.5 | -18.4 | 7.8 | 2.7 | 2.6 |
| 5/10/2013 | *Manta birostris* | Male | 3.9 | -19.2 | -18.7 | 8 | 2.8 | 2.7 |
| 6/10/2013 | *Manta birostris* | Male | 3.8 | -19.5 | -19.1 | 7.3 | 2.6 | 2.4 |
| 24/08/2014 | *Manta birostris* | Female | 2.7 | -14.5 | -14.5 | 9.2 | 3.1 | 3.2 |
| 25/08/2014 | *Manta birostris* | Female | 3.6 | -17.9 | -17.7 | 10.1 | 3.3 | 3.6 |
| 26/08/2014 | *Manta birostris* | Female | 3.4 | -16.7 | -16.7 | 10 | 3.3 | 3.6 |
| 26/08/2014 | *Manta birostris* | Female | 3.5 | -16.8 | -16.8 | 9.9 | 3.3 | 3.5 |
| 26/08/2014 | *Manta birostris* | Female | 3.7 | -17.1 | -16.8 | 9.1 | 3.1 | 3.2 |
| 28/08/2014 | *Manta birostris* | Female | 3.5 | -17.6 | -17.6 | 9 | 3.0 | 3.1 |
| 28/08/2014 | *Manta birostris* | Female | 3.4 | -16.5 | -16.5 | 8.1 | 2.8 | 2.7 |
| 4/09/2014 | *Manta birostris* | Female | 3.6 | -16.7 | -16.5 | 10.8 | 3.5 | 3.9 |
| 6/09/2014 | *Manta birostris* | Female | 3.4 | -17.6 | -17.6 | 7.7 | 2.7 | 2.6 |
| 9/09/2014 | *Manta birostris* | Female | 4.5 | -17.1 | -16 | 7.6 | 2.7 | 2.5 |
| 12/09/2014 | *Manta birostris* | Female | 3.5 | -17.2 | -17.2 | 9 | 3.0 | 3.1 |
| 24/08/2014 | *Manta birostris* | Male | 2.8 | -14.7 | -14.7 | 10.3 | 3.4 | 3.7 |
| 25/08/2014 | *Manta birostris* | Male | 3.5 | -17.6 | -17.6 | 8.8 | 3.0 | 3.0 |
| 1/09/2014 | *Manta birostris* | Male | 3.4 | -17.4 | -17.4 | 8.1 | 2.8 | 2.7 |
| 1/09/2014 | *Manta birostris* | Male | 3.4 | -16.6 | -16.6 | 9.9 | 3.3 | 3.5 |
| 9/09/2014 | *Manta birostris* | Male | 3.5 | -17.6 | -17.6 | 7.7 | 2.7 | 2.6 |
| 10/09/2014 | *Manta birostris* | Male | 3.7 | -17.7 | -17.4 | 8.6 | 2.9 | 2.9 |
| 12/09/2014 | *Manta birostris* | Male | 3.3 | -16.8 | -16.8 | 9.4 | 3.1 | 3.3 |
| 13/09/2014 | *Manta birostris* | Male | 3.4 | -17.4 | -17.4 | 9.1 | 3.1 | 3.2 |
| 27/08/2014 | *Manta birostris* | Male | 3.6 | -17.8 | -17.6 | 10.2 | 3.4 | 3.6 |
| 6/09/2014 | *Manta birostris* | Female | 3.3 | -16.9 | -16.9 | 10.7 | 3.5 | 3.9 |
| 4/09/2014 | *Manta birostris* | Female | 3.7 | -18.1 | -17.8 | 10.6 | 3.5 | 3.8 |

*δ^13^C values only normalised when C:N ratios were >3.5 [13]

Table S2. C:N, δ^13^C and δ^15^N values for zooplankton tows

| Date Sampled | Species | C:N | δ^13^C | δ^13^C _normalised_ | δ^15^N |
| --- | --- | --- | --- | --- | --- |
| 31/08/2013 | Zooplankton | 3.4 | -19.9 | -19.9 | 9.7 |
| 31/08/2013 | Zooplankton | 3.7 | -20.5 | -20.7 | 8.4 |
| 1/10/2013 | Zooplankton | 3.8 | -20.4 | -20.4 | 7.6 |
| 1/10/2013 | Zooplankton | 3.9 | -20.6 | -20.4 | 8.2 |
| 2/10/2013 | Zooplankton | 4.1 | -20.7 | -21.3 | 9 |
| 2/10/2013 | Zooplankton | 4 | -20.8 | -20.4 | 9.5 |
| 6/10/2013 | Zooplankton | 4.4 | -20.4 | -19.3 | 8.6 |
| 6/10/2013 | Zooplankton | 4.6 | -20.6 | -19.2 | 8.5 |
| 26/08/2013 | Zooplankton | 3.8 | -21 | -21 | 7.9 |
| 26/08/2013 | Zooplankton | 4.6 | -20.8 | -19.4 | 8.1 |
| 29/08/2013 | Zooplankton | 3.9 | -20.8 | -20.6 | 8.9 |
| 3/09/2013 | Zooplankton | 3.9 | -20.8 | -20.6 | 8.2 |
| 3/09/2013 | Zooplankton | 4.3 | -21.1 | -20.2 | 8.9 |
| 3/09/2013 | Zooplankton | 3.7 | -20.4 | -20.6 | 7.8 |
| 3/09/2013 | Zooplankton | 3.5 | -20.2 | -20.2 | 8.1 |
| 12/09/2013 | Zooplankton | 3.8 | -21.1 | -21.1 | 8.3 |
| 12/09/2013 | Zooplankton | 3.8 | -22.1 | -22.1 | 7.5 |
| 19/09/2013 | Zooplankton | 4.4 | -21.1 | -20 | 8.2 |
| 4/10/2013 | Zooplankton | 3.9 | -18.6 | -18.4 | 8.8 |
| 23/08/2014 | Zooplankton | 4.9 | -20.2 | -18.4 | 5.5 |
| 24/08/2014 | Zooplankton | 4.4 | -21 | -19.9 | 6 |
| 27/08/2014 | Zooplankton | 4.3 | -20.2 | -19.3 | 5.8 |
| 1/09/2014 | Zooplankton | 5.3 | -20.1 | -17.9 | 6.8 |
| 4/09/2014 | Zooplankton | 4.7 | -19.7 | -18.2 | 7.3 |
| 9/09/2014 | Zooplankton | 4.5 | -19.2 | -18 | 7 |
| 18/09/2014 | Zooplankton | 5.2 | -20.2 | -18.1 | 6.5 |
| 21/09/2014 | Zooplankton | 4.7 | -20.6 | -19.1 | 7 |
| 21/09/2014 | Zooplankton | 4.5 | -20.3 | -19.1 | 6.8 |
| 30/09/2014 | Zooplankton | 4.6 | -21 | -19.6 | 6.1 |
| 10/09/2014 | Zooplankton | 4.6 | -19.6 | -18.2 | 7.9 |
| 12/09/2014 | Zooplankton | 4.3 | -20 | -19.1 | 8 |
| 14/09/2014 | Zooplankton | 4.8 | -20.9 | -19.2 | 8.1 |
| 24/09/2014 | Zooplankton | 4.7 | -21 | -19.5 | 8.3 |
| 26/09/2014 | Zooplankton | 4.7 | -20.7 | -19.2 | 7.8 |
| 29/09/2014 | Zooplankton | 4.7 | -20.9 | -19.4 | 7.9 |

Table S3. Mixing model summary statistics on the mean credible interval source contributions of surface zooplankton and mesopelagic sources to *M. birostris* diet. Model 1 source inputs comprised of mesopelagic fishes and lipid normalised surface zooplankton δ^13^C values and used DTDFs from large sharks [16]. Model 2 source inputs were mesopelagic fishes and non-lipid normalised surface zooplankton δ^13^C with the large shark DTDF [16]. Models 3 and 4 comprised the same source inputs as model 1 and 2 respectively, but used DTDF values from T. semifasciata [17]. Model 5 used the highest δ^13^C and δ^15^N values of surface zooplankton and mesopelagic fishes to assess whether extreme surface zooplankton values could account for the discrepancy in stable isotope profiles between surface zooplankton and *M. birostris*.

| **Model** | **Source** | **2.5%** | **25%** | **50%** | **75%** | **97.5%** |
| --- | --- | --- | --- | --- | --- | --- |
| 1 | Surface Zooplankton | 0.037 | 0.086 | 0.115 | 0.147 | 0.215 |
|  | Mesopelagic fishes | 0.785 | 0.853 | 0.885 | 0.914 | 0.963 |
|  | s.d. δ^13^C | 0.468 | 0.684 | 0.787 | 0.893 | 1.109 |
|  | s.d. δ^15^N | 1.578 | 1.875 | 2.042 | 2.219 | 2.593 |
|  |  |  |  |  |  |  |
| 2 | Surface Zooplankton | 0.077 | 0.154 | 0.204 | 0.254 | 0.363 |
|  | Mesopelagic fishes | 0.637 | 0.746 | 0.796 | 0.846 | 0.923 |
|  | s.d. δ^13^C | 0.564 | 0.764 | 0.866 | 0.976 | 1.209 |
|  | s.d. δ^15^N | 1.548 | 1.821 | 1.977 | 2.152 | 2.527 |
|  |  |  |  |  |  |  |
| 3 | Surface Zooplankton | 0.321 | 0.393 | 0.431 | 0.468 | 0.541 |
|  | Mesopelagic fishes | 0.459 | 0.532 | 0.569 | 0.607 | 0.679 |
|  | s.d. δ^13^C | 0.558 | 0.713 | 0.798 | 0.88 | 1.064 |
|  | s.d. δ^15^N | 0.701 | 0.93 | 1.055 | 1.177 | 1.434 |
| 4 | Surface Zooplankton | 0.241 | 0.298 | 0.327 | 0.357 | 0.411 |
|  | Mesopelagic fishes | 0.589 | 0.643 | 0.673 | 0.702 | 0.759 |
|  | s.d. δ^13^C | 0.598 | 0.753 | 0.833 | 0.925 | 1.104 |
|  | s.d. δ^15^N | 0.495 | 0.814 | 0.96 | 1.092 | 1.358 |
|  |  |  |  |  |  |  |
| 5 | Surface Zooplankton ^13^C* | 0.050 | 0.197 | 0.314 | 0.417 | 0.567 |
|  | Surface Zooplankton ^15^N** | 0.026 | 0.081 | 0.137 | 0.204 | 0.303 |
|  | Mesopelagic fishes*** | 0.373 | 0.489 | 0.549 | 0.608 | 0.695 |
|  | s.d. δ^13^C | 0.785 | 0.927 | 1 | 1.076 | 1.237 |
|  | s.d. δ^15^N | 0.304 | 0.819 | 1.008 | 1.162 | 1.456 |

* δ^13^C = -18.6, δ^15^N = 8.8

** δ^13^C = -19.9, δ^15^N = 9.7

*** δ^13^C = -17, δ^15^N = 7

In Model 5 (table S3), surface zooplankton with enriched δ^13^C was found on average to contribute 31.3 ± 14.3 % to the diet of *M. birostris*, while surface zooplankton with enriched δ^15^N contributed 14.2 ± 7.8 % and mesopelagic fishes contributed 54.4 ± 8.3 %.

**Supplementary References**

Ménard F, Lorrain A, Potier M, Marsac F. 2007 Isotopic evidence of distinct feeding ecologies and movement patterns in two migratory predators (yellowfin tuna and swordfish) of the western Indian Ocean*.* *Mar. Biol.* **153**, 141–152. (doi: 10.1007/s00227-007-0789-7)

16. Choy CA, Popp BN, Hannides C, Drazen JC. 2015 Trophic structure and food resources of epipelagic and mesopelagic fishes in the North Pacific Subtropical Gyre ecosystem inferred from nitrogen isotopic compositions*.* *Limnol. Oceanogr.* **60**, 1156–1171. (doi:10.1002/lno.10085)

17. Hussey NE, Brush J, McCarthy ID, Fisk AT. 2010 δ15N and δ13C diet–tissue discrimination factors for large sharks under semi-controlled conditions*.* *Comp. Biochem. Physiol. A Mol. Integr. Physiol.* **155**, 445–453. (doi:10.1016/j.cbpa.2009.09.023)

26. Kelez Sara S. 2011 Bycatch and Foraging Ecology of Sea Turtles in the Eastern Pacific, PhD Thesis, in *Department of Environment* Duke University, North Carolina, USA: Ann Arbor.

28. Polo-Silva C, Newsome SD, Galván-Magaña F, Grijalba-Bendeck M, Sanjuan-Muñoz A. 2013 Trophic shift in the diet of the pelagic thresher shark based on stomach contents and stable isotope analyses*.* *Mar. Biol. Res.* **9**, 958–971. (doi:10.1080/17451000.2013.793802)
